# Protocol for doping of an Sn-based two-dimensional perovskite semiconductor by incorporating SnI_4_ for field-effect transistors and thermoelectric devices

**DOI:** 10.1016/j.xpro.2022.101876

**Published:** 2022-11-19

**Authors:** Yu Liu, Ping-An Chen, Xincan Qiu, Jing Guo, Jiangnan Xia, Huan Wei, Haihong Xie, Shijin Hou, Mai He, Xiao Wang, Zebing Zeng, Lang Jiang, Lei Liao, Yuanyuan Hu

**Affiliations:** 1 Key Laboratory for Micro/Nano Optoelectronic Devices of Ministry of Education & International Science and Technology Innovation Cooperation Base for Advanced Display Technologies of Hunan Province, School of Physics and Electronics, Hunan University, Changsha 410082, China; 2 Shenzhen Research Institute of Hunan University, Shenzhen 518063, China; 3 State Key Laboratory of Chemo/Biosensing and Chemometrics, College of Chemistry and Chemical Engineering, Hunan University, Changsha 410082, China; 4 Beijing National Laboratory for Molecular Sciences, Key Laboratory of Organic Solids, Institute of Chemistry, Chinese Academy of Sciences, Beijing 100190, China

**Keywords:** Physics, Material sciences

## Abstract

Doping is an important technique for semiconductor materials, yet effective and controllable doping of organic-inorganic halide perovskites is still a challenge. Here, we present a protocol to dope 2D perovskite (PEA)_2_SnI_4_ by incorporating SnI_4_ in the precursor solutions. We detail steps for preparation of field-effect transistors (FETs) and thermoelectric devices (TEs) based on SnI_4_-doped (PEA)_2_SnI_4_ films. We further describe characterization via conductivity measurement using the four-point probe method, FETs performance, and TEs performance measurements.

For complete details on the use and execution of this protocol, please refer to Liu et al. (2022).^1^

## Highlights

• Detailed procedures for fabrication of Sn^4+^-doped PEA_2_SnI_4_ FETs and TEs
• Conductivity measurement of doped-PEA_2_SnI_4_ films by four-point probe method
• Characterization of the performance of doped-PEA_2_SnI_4_ FET devices
• Characterization of the Seebeck coefficients and power factors of doped-PEA_2_SnI_4_ TEs

## Abstract (editor highlights)

Publisher’s note: Undertaking any experimental protocol requires adherence to local institutional guidelines for laboratory safety and ethics.

## Graphical abstract

**Figure:**
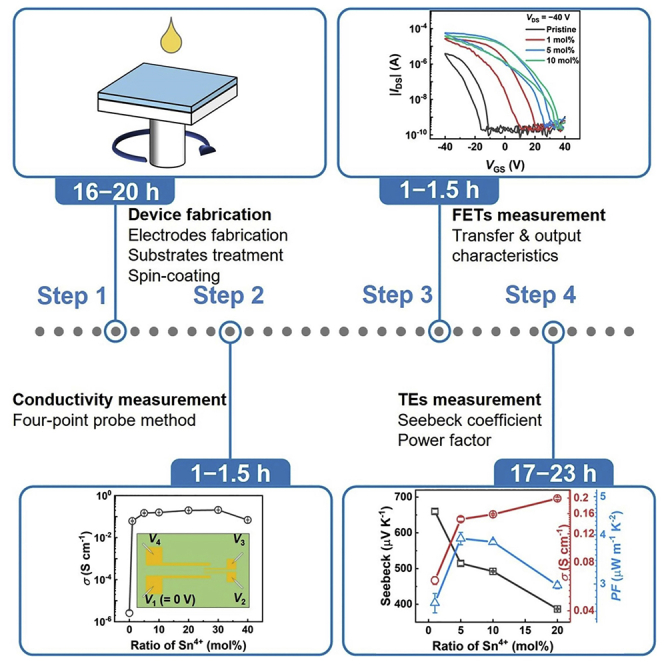

## Before you begin

**Timing: 2–4 h**

The protocol below describes the specific steps for preparing and characterizing field-effect transistors (FETs) and thermoelectric devices (TEs) based on SnI_4_-doped (PEA)_2_SnI_4_ films.

1. Check the oxygen and moisture level in the glovebox, it should be approximately or less than 0.1 ppm of H_2_O and 0.1 ppm of O_2_ level.
2. The important materials for making (PEA)_2_SnI_4_ including phenethylammonium iodide, Tin(II) iodide and Tin(IV) iodide, etc. listed in the key resources table, should be stored in the glovebox preferably for less than three months.

## Key resources table

**Table:** 

REAGENT or RESOURCE	SOURCE	IDENTIFIER
**Chemicals, peptides, and recombinant proteins**		

Phenethylammonium iodide, ≥99.5%	Xi’an Polymer	Cat#PLT501391I
Tin(II) iodide, AnhydroBeads™, −10 mesh, 99.99% trace metals basis	Aldrich	Cat#409308
*N,N*-Dimethylformamide, anhydrous, 99.8%	Aldrich	Cat#227056
1-Methyl-2-pyrrolidinone, anhydrous, 99.5%, packaged under argon in resealable ChemSealTM bottles	Alfa Aesar	Cat#043741
Tin(IV) iodide, 95%	Aladdin	Cat#T195042
Octadecyltrichlorosilane, 95%	Acros	Cat# 147400250
Toluene, AR	Innochem	Cat# I00975
Chrome rod, 99.99%	Kurt J. Lesker Co., Ltd	N/A
Gold grain, 99.99%	Hezong Xincai Technology Co., Ltd	N/A
Glass substrate	Luoyang Guluoglass Co., Ltd	N/A
Si/SiO_2_ substrate	Suzhou EDMICRO Technology Co., Ltd	N/A
Scotch magic tape	3M Technology Co., Ltd	Cat# 810-CQ33
Sodium hydroxide (NaOH), AR, 99%	Innochem	Cat# A36865
Acetone, AR, 99%	Innochem	Cat# 12378
Isopropanol, AR, 99%	Innochem	Cat# A17203
Positive photoresist	Kempur Microelectronics Inc.	Cat# BP212-37S

**Other**		

Keithley 4200 semiconductor analyzer	Tektronix Technologies	https://www.tek.com/en/keithley-4200a-scs-parameter-analyzer
B2912A Precision Source	Keysight	https://www.keysight.com/us/en/product/B2912A/precision-source-measure-unit-2-ch-10fa-210v-3a-dc-10-5a-pulse.html?rd=1
ST-100 cryostat	Janis	https://www.lakeshore.com/products/product-detail/janis/st-100-optical-cryostat
Model 22C temperature controller	Cryo-con	https://www.cryocon.com/M22CProdFolder.php
Keithley nano voltmeter model 2182A	Tektronix Technologies	https://www.tek.com.cn/products/keithley/low-level-sensitive-and-specialty-instruments/nanovoltmeter-model-2182a
DC stabilized power supply model DP152	Mestek	http://www.china-nengyuan.com/product/180303.html
Single-sided lithography machine model H19-13	Sichuan Hongyuan Dingxin Technology Co., Ltd	http://www.schydx.com/pd.jsp?id=33#_pp=2_748
Evaporator model PD-400S	Wuhan PDVACUUM Technology Co., Ltd	http://www.pdvacuum.com/index.php?m=content&c=index&a=show&catid=7&id=61
UV/ozone cleaner	Sunmonde Technology Co., Ltd	http://www.sunmonde.com.cn/
Spin-coater	LEBO science Technology Co., Ltd	http://www.leboscience.cn/view/id/85.html
Analytical balance, model BSA124S	Sartorius Technology Co., Ltd	https://www.sartorius17.cn/bsatp/0.1mgfxtp/bsa124s.html
Glovebox, model Universal	MIKROUNA Technology Co., Ltd	https://www.mikrouna.com/product/index/id/37.html

## Step-by-step method details

### Device fabrication

**Timing: 16–20 h**

**Timing: 5–7 h (for step 1a**–**1e)**

**Timing: 10–11.5 h (for step 2)**

**Timing: 1–1.5 h (for step 3)**

The preparation process includes preparing perovskite precursor solutions and spin-coating precursor solutions on cleaned substrates.

**Figure 1:**
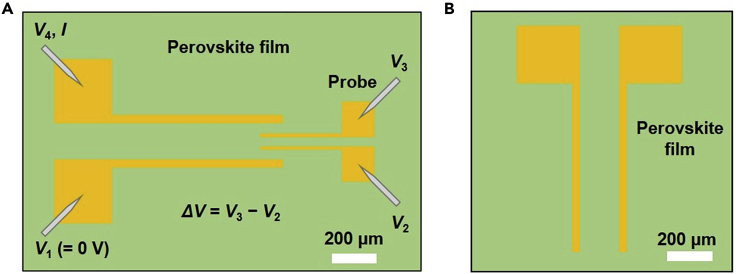
Electrode structures of devices Scale bar: 200 μm. (A) Electrode structure of conductivity measurement. (B) Electrode structure of FET measurement.
  c. For FET devices, Figure 1B shows the electrode structure. Deposit Cr (2 nm) and Au (30 nm) sequentially on Si^++^/SiO_2_ substrates by a thermal evaporator.
  d. For TE devices, Figure 2A shows the electrode structure. Deposit Cr (10 nm) and Au (15 nm) sequentially on glass substrates by a thermal evaporator.

1. Bottom-contact electrodes fabrication.
  Define and deposit the bottom-contact electrodes by photolithography and thermal evaporator (< 4 × 10^-4^ Pa), respectively.
  a. Define bottom-contact electrodes by photolithography.
    i. Dissolve 2 g NaOH with 500 mL deionized water as developer.
    ii. Spin the positive photoresist on the desired 4-inch wafer substrate with speed of 3,000 rpm for 30 s and anneal at 105°C for 5 min.
    iii. Expose the substrate under UV light through shadow mask for 7 s.
    iv. Soak the substrate into the developer for 10 s, and dry them with nitrogen gas gun.
  b. For conductivity measurement, Figure 1A shows the electrode structure. Deposit the Cr (2 nm) and Au (30 nm) sequentially on Si^++^/SiO_2_ substrates by a thermal evaporator.

**Figure 2:**
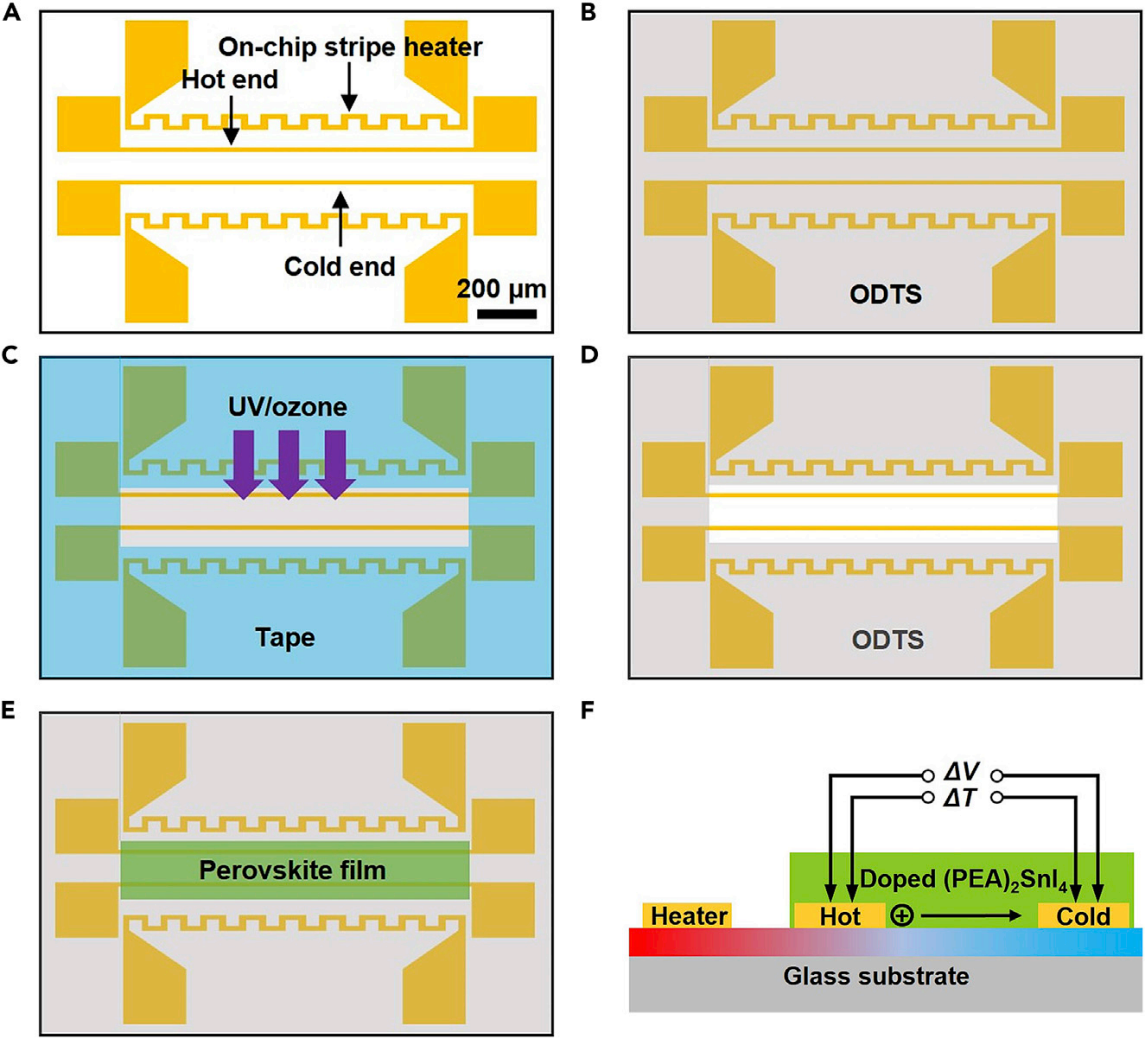
Fabrication process and measurement schematic of TE devices Scale bar: 200 μm. (A)–(E) Fabrication process of TE devices. (F) Measurement schematic of TE devices. This figure is adopted from ref.^1^.
  e. Soak the substrates into acetone for 30 min to lift off the photoresist, and dry them with nitrogen gas gun.
    ***Note:*** For Si^++^/SiO_2_ substrates, Si^++^ refers to heavily doped silicon with low resistivity of 0.01 Ω/cm. The thickness of SiO_2_ is 300 nm.

2. Preparing perovskite precursor solutions.
  a. Weigh SnI_2_ (0.1 mmol, 37.3 mg) and PEAI (0.2 mmol, 49.8 mg) in a 1.5 mL glass reagent bottle in sequence.
    i. Add 250 μL 1-Methyl-2-pyrrolidinone (NMP) and 750 μL dimethyl formamide (DMF) into the reagent bottle to form solution A (0.1 M).
  b. Weigh SnI_4_ (0.1 mmol, 62.6 mg) and PEAI (0.2 mmol, 49.8 mg) in a 1.5 mL glass reagent bottle in sequence.
    i. Add 250 μL NMP and 750 μL DMF into the reagent bottle to form solution B (0.1 M).
  c. Mix solution A and solution B with volume ratio of 1-*x*:*x*, where *x* represents the doping ratio of SnI_4_ to obtain 0.1 M (PEAI)_2_(SnI_2_)_1-*x*_(SnI_4_)_*x*_ precursor solutions.
  d. After heating the (PEAI)_2_(SnI_2_)_1-*x*_(SnI_4_)_*x*_ precursor solutions at 60°C for 9 h.
    i. Store the solutions at room temperature (about 25°C here) for 1 h to cool down.
    ii. Filter solutions through 0.45 μm PTFE filters.

**CRITICAL:** The standard mass ratio of SnI_2_ and PEAI should be 0.748. The standard mass ratio of SnI_4_ and PEAI should be 1.257.

**CRITICAL:** The prepared precursor solutions should not be stored for more than 2 days.

3. Substrate cleaning and spin coating.
  a. Cut the 4-inch wafer substrate into pieces (about 1 cm × 1.5 cm).
  b. For conductivity and FET measurements, ultrasonicate the Si^++^/SiO_2_ substrates sequentially in deionized water, acetone and isopropanol for 2 min each.
    i. Blow dry them by a nitrogen gas gun.
    ii. Treat the substrates with UV/ozone for 30 min before spin-coating.
    iii. Cast 30 μL precursor solution in the center of the Si^++^/SiO_2_ substrate and spin-coat at 4,000 rpm for 30 s with acceleration of 500 rpm/s.
    iv. Anneal the perovskite films at 100°C for 10 min.
  c. For TEs measurement, pattern the perovskite films as shown in Figure 2.
    i. Ultrasonicate the glass substrates sequentially in deionized water, acetone and isopropanol for 2 min each.
    ii. Blow dry them by a nitrogen gas gun.
    iii. Treat the substrates with UV/ozone for 30 min.
    iv. Spin Octadecyltrichlorosilane (ODTS) solution (5 vol‰ in toluene) on the glass substrates with at 2,000 rpm for 30 s with acceleration of 1,000 rpm/s.
    v. Anneal the substrates at 100°C for 10 min to form an ODTS film on substrate (Figure 2B).
    vi. Cover the area except for the hot and cold ends with tape.
    vii. Treat the substrates with UV/ozone for 30 min (Figure 2C) before removing the tape (Figure 2D).
    viii. Cast 10 μL precursor solution in the center of the glass substrate and spin-coat at 4,000 rpm for 30 s with acceleration of 500 rpm/s.
    ix. Anneal the perovskite films at 100°C for 10 min to form patterned perovskite film (Figure 2E).

**CRITICAL:** The area covered by ODTS is hydrophobic, thus the perovskite film can’t form on ODTS covered area.

**CRITICAL:** The time from the end of UV/ozone treatment to the beginning of spin coating should be less than 5 min to avoid weakening of the hydrophilicity. Complete all processes in the glovebox except for the substrate cleaning.

### Device characterization

**Timing: 17–23 h**

**Timing: 1–1.5 h (for step 4)**

**Timing: 1–1.5 h (for step 5)**

**Timing: 15–20 h (for step 6)**

The characterizations include conductivity, FETs performance, and TEs performance measurements. Due to the instability of perovskite films, conduct all measurements immediately in Ar-filled glovebox in dark after preparation unless otherwise stated.

4. Conductivity measurement.
  Measure the conductivity of perovskite film by four-point probe method, Figure 1A shows the electrode structure.
  a. Connect the four electrodes with a Keithley 4200 semiconductor analyzer using a probe station.
  b. As shown in Figure 1A, the voltages of the four electrodes are named *V*_1_ (= 0 V), *V*_2_, *V*_3_ and *V*_4_, respectively.
  c. After the four-point probe measurement, identify the thicknesses of samples by atomic force microscopy (AFM).
    **CRITICAL:** The measured *V*_4_ should be more than 1 V and less than 20 V by adjusting the maximum value of sweep current *I*.

5. FETs measurement.
  Figure 1B shows the electrode structure of bottom-gate bottom-contact FET. Measure the FETs using a B2912A Precision Source.
  a. For room-temperature (about 25°C here) measurement, place the samples on the insulated stage of probe station in glovebox.
  b. Connect the FETs with a B2912A Precision Source using a probe station in glovebox.
  c. Measure the transfer characteristics.
    i. Set the gate voltage (*V*_GS_) sweeps from 40 V to –40 V and back to 40 V (step is –1 V, sweep speed is 25 V s^-1^).
    ii. Set the drain voltage (*V*_DS_) as –40 V.
  d. Measure the output characteristics.
    i. Set the *V*_DS_ sweeps from 0 V to –40 V and back to 0 V (step is –1 V, sweep speed is 20 V s^-1^).
    ii. Set the *V*_GS_ as 0, –20 and –40 V, respectively.
      **CRITICAL:** Carry out all measurement processes under dark conditions.
      **CRITICAL:** Conduct the transfer and output characteristics twice, and save the result of the second measurement, because the light exposure before the test will affect the results of the first measurement.

6. TEs measurement.
  To measure the Seebeck coefficients of doped (PEA)_2_SnI_4_ films, we use a homemade thermoelectric measurement system, as shown in Figure 2A.^2^
  a. Connect the pads of on-chip stripe heater, hot end and cold end with probes of Janis ST-100 cryostat in an Ar-filled glovebox.
  b. Close the exhaust valve of the cryostat before transferring it out from the glovebox to protect perovskite from the invasion of air.
  c. Measure the resistances (*R*) of hot end and cold end under 302, 304 and 306 K using B2912A Precision Source and temperature controller.
  d. Link the on-chip stripe heater to a DC stabilized power supply with external wires, and apply a voltage (*V*_heater_) to the heater.
  e. Change *V*_heater_ from 4 V to 10 V (step = 1 V).
    i. Measure the corresponding *ΔV* using Keithley nano voltmeter.
    ii. Measure the resistances of hot end and cold end by B2912A Precision Source.
      ***Note:*** The TEs measurement aims to obtain the relationship between temperature difference (*ΔT*) and thermoelectric potential difference (*ΔV*), as shown in Figure 2F.
      **CRITICAL:** Measure all Seebeck coefficients at RT in a high vacuum (< 10^–5^ mbar) using Janis ST-100 in the dark.

## Expected outcomes

**Figure 3:**
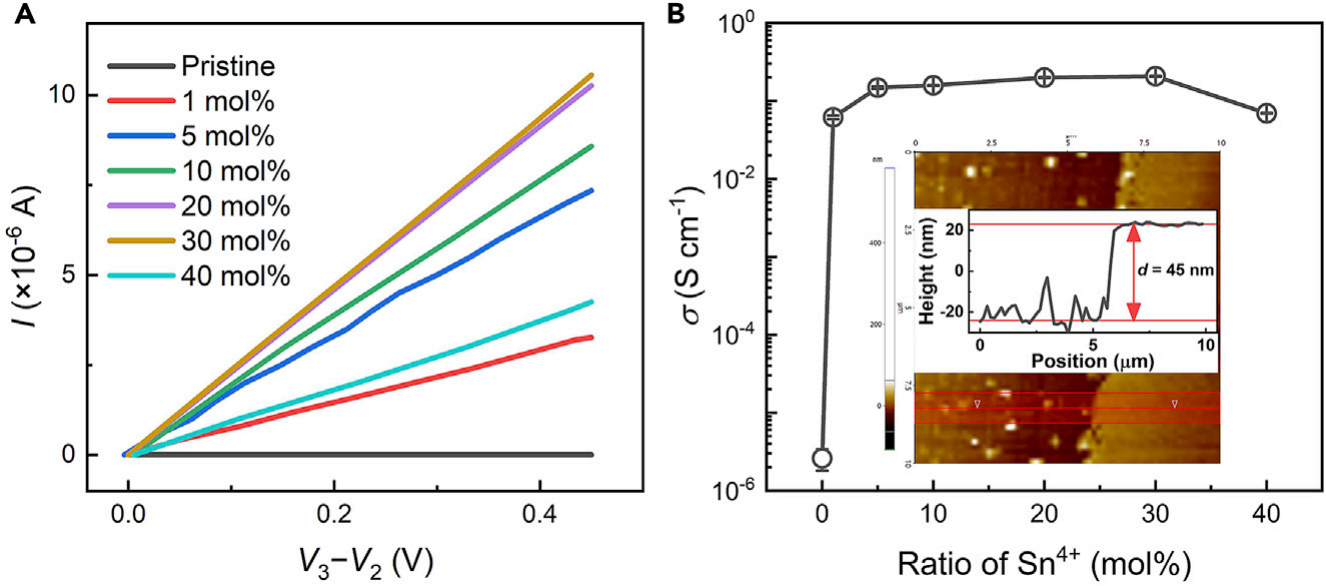
Outcomes of conductivity measurement Figures are adopted with changes from ref.^1^. (A) Current-voltage curves of perovskite films with different SnI_4_ ratios. (B) Electrical conductivities as a function of doping ratio calculated from (A). The error bars represent standard deviation. Inset: thickness of film identified by AFM measurement.

The important outcomes of the present protocols are illustrated below:

**Conductivity measurement:** The relationship of *I* and *V*_3_ *– V*_2_ of doped perovskite films can be obtained by four-point probe method (see Figure 3A), and the slope is the conductance (*G*) of perovskite film. The conductivity *σ* [S cm^-1^] = 40 × *G* [S]/ (1000 × *d* [nm] ×10^-7^), *d* (∼45 nm) is the thickness of film which is identified by AFM. The doping ratio-dependent conductivity is shown in Figure 3B which directly demonstrates the occurrence of doping.

**Figure 4:**
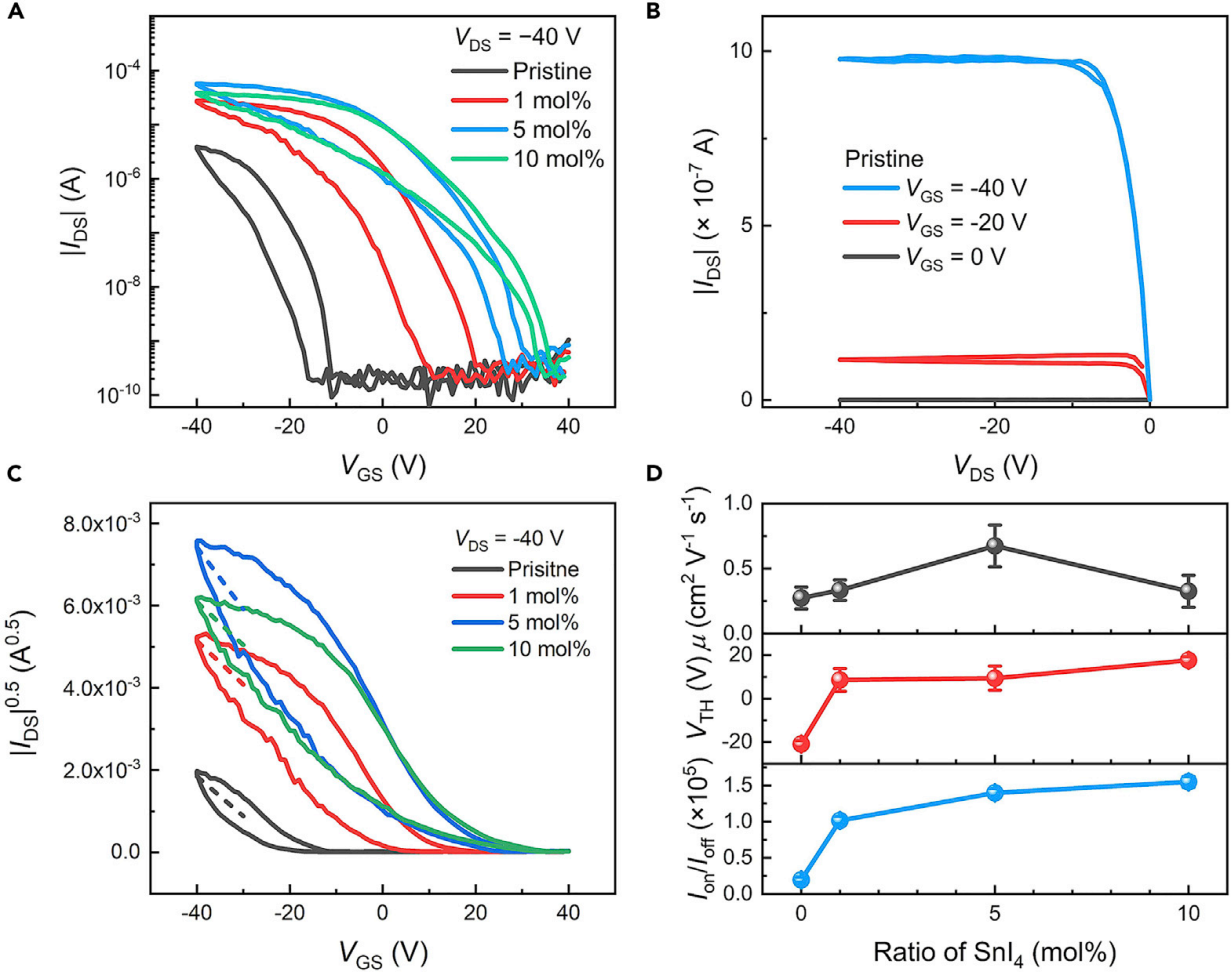
Outcomes of FETs measurement Figures are adopted from ref.^1^. (A) Transfer characteristics of FETs based on doped (PEA)_2_SnI_4_ films. (B) Output characteristics of FETs based on pristine (PEA)_2_SnI_4_ films. (C) *I*_DS_^0.5^ versus *V*_GS_ curves calculated from (A), dash lines are linearly fitted lines. (D) Extracted mobility, threshold voltage and on/off ratio from (A) and (C). The error bars represent standard deviation.

**FETs measurement:** The measured transfer and output characteristics of FETs based on doped (PEA)_2_SnI_4_ films are shown in Figure 4A and 4B. The on/off ratio (*I*_on_/*I*_off_) can be extracted from Figure 4A, *I*_on_ and *I*_off_ are on-state and off-state currents, which are the maximum and minimum currents of transfer curves, respectively. Here, the *I*_on_ and *I*_off_ are corresponding to the currents at *V*_GS_ of 40 V and −40 V, respectively. According to the *I*_DS_^0.5^ versus *V*_GS_ curves (Figure 4C), the mobility (*μ*) and threshold voltage (*V*_TH_) can be extracted from Figure 4C according to the equations $\mu =\frac{2L}{C_{\text{i}}W}\left(\frac{\partial {I_{\text{DS}}}^{0.5}}{\partial V_{\text{GS}}}\right)$ and $I_{\text{DS}}=C_{\text{i}}\mu \frac{W}{2L}{\left(V_{\text{GS}}-V_{\text{TH}}\right)}^{2}$, respectively.^3^ *L* and *W* are length and width of the FETs channel, respectively. The fitted range of *V*_GS_ is from −40 V to −30 V. The doping ratio-dependent performance parameters are shown in Figure 4D.

**Figure 5:**
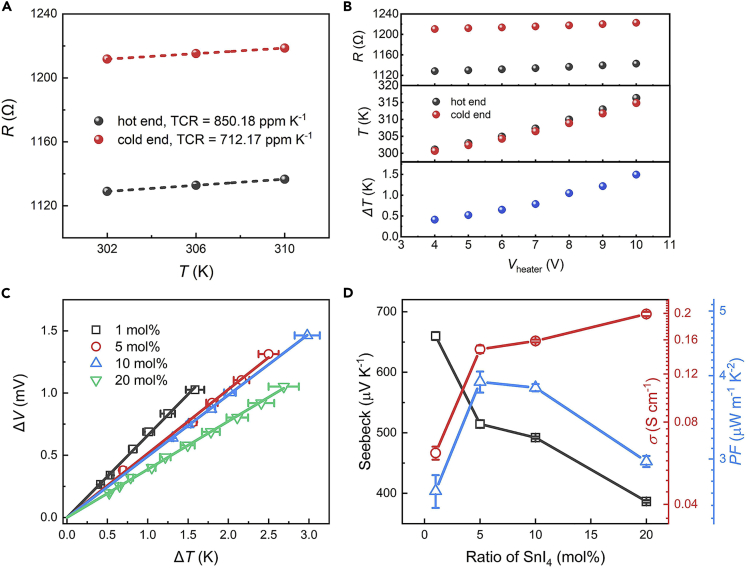
Outcomes of TEs measurement Figures C and D are adopted from ref.^1^. (A) Temperature-dependent resistance of hot end and cold end. (B) Heater voltage-dependent resistance, calculated temperature and temperature difference. (C) Temperature difference (*ΔT*)-dependent thermoelectric potential difference (*ΔV*) of doped TEs. (D) Doping ratio-dependent Seebeck coefficient, conductivity and power factor. The error bars represent the standard deviation.

**TEs measurement:** The measured temperature-dependent resistances of hot end and cold end are shown in Figure 5A. Temperature coefficient of resistance (TCR) can be extracted by formula: TCR [K^-1^] = d*R*/(R_302K_d*T*). *R* and *T* are real-time resistance and temperature, respectively. As shown in Figure 5B, resistances increase with increased *V*_heater_, the corresponding temperatures of hot end and cold end can be calculated according to the equation $T=\text{302}+\frac{R_{\text{T}}-R_{302\text{K}}}{\text{TCR}\times R_{302\text{K}}}$, *R*_T_ is the measured resistance at temperature of *T*, and *R*_302K_ is the measured resistance at 302 K. The temperature difference between hot end and cold end can also be calculated (Figure 5B). The measured *ΔT* versus *ΔV* is shown in Figure 5C, the slope of the fitted line is the Seebeck coefficient (*S* = *ΔV*/*ΔT*). The power factor (*PF* = *S*^2^*σ*) can be calculated from *S* (Figure 5C) and *σ* (Figures 3B),^4^ these parameters are shown in Figure 5D.

## Limitations

The conductivity of pristine (PEA)_2_SnI_4_ film is too low to measure the Seebeck coefficient accurately.

## Troubleshooting

### Problem 1

The measured conductivity is much higher than the expected value (device characterization step 4).

### Potential solution

The much higher conductivity is contributed to the oxidation of Sn^2+^. The possible reasons are that SnI_2_, perovskite solution or device is stored too long.

Solution 1: Use newly purchased SnI_2_.

Solution 2: Prepare new solution.

Solution 3: Measure the devices as soon as possible after fabrication.

### Problem 2

The on/off ratio of transfer characteristics is much lower than the expected value (device characterization step 5).

### Potential solution

The much lower on/off ratio may be caused by that the actual mass ratio differs greatly from the standard value.

Solution 1: Weigh SnI_2_ and PEAI with mass ratio of 0.748 strictly.

Solution 2: Weigh SnI_4_ and PEAI with mass ratio of 1.257 strictly.

### Problem 3

The Seebeck coefficient of TE does not decrease with the increase of conductivity, or the resistance versus temperature of hot end and cold end (Figure 5A) is not perfectly linear (device characterization step 6).

### Potential solution

Solution 1: Reconnect the probes to the electrodes.

Solution 2: Wait 20 min for the temperature to be stable before measuring resistance.

### Problem 4

Imperfect perovskite film deposition may be attributed to the dirty atmosphere in glovebox (such as the existence of organic solvent vapor) or the temperature of precursor is much higher than room temperature (about 25°C here) (device fabrication step 3).

### Potential solution

Solution 1: Clean the glovebox with fresh Ar gas for at least 10 min.

Solution 2: Place the precursor away from heat for half an hour.

### Problem 5

No/flawed measurement (FET measurement) (device characterization step 5).

### Potential solution

Solution 1: If the currents of transfer or output measurements are very small, i.e., no FETs current, check the connection between probes and electrodes, and connection between probe station and Precision Source.

Solution 2: If the currents of transfer or output curves are much higher than the typical curves (as shown in Figure 4A and 4B), check that if the measurement environment is dark.

## Resource availability

### Lead contact

Further information and requests for resources and reagents should be directed to and will be fulfilled by the lead contact, Yuanyuan Hu (yhu@hnu.edu.cn).

### Materials availability

This study did not generate new unique reagents.

## Data Availability

This study did not produce datasets/code.
